# Effectiveness of Technology-Enabled, Low Carbohydrate Dietary Interventions, in the Prevention or Treatment of Type 2 Diabetes Mellitus in Adults: A Systematic Literature Review of Randomised Controlled and Non-Randomised Trials

**DOI:** 10.3390/nu15204362

**Published:** 2023-10-13

**Authors:** Bernice Rozemai Jooste, Despina Kolivas, Peter Brukner, George Moschonis

**Affiliations:** School of Allied Health, Human Services & Sport, La Trobe University, Bundoora, VIC 3086, Australia; 18790557@students.latrobe.edu.au (B.R.J.); d.kolivas@latrobe.edu.au (D.K.); p.brukner@latrobe.edu.au (P.B.)

**Keywords:** type 2 diabetes, prediabetes, low carbohydrate diets, technology, m-health, glycosylated haemoglobin

## Abstract

Evidence suggests that low carbohydrate dietary (LCD) approaches can improve glycaemic control and may result in type 2 diabetes mellitus (T2DM) remission. This systematic literature review (SLR) aimed to assess the effectiveness of technology-enabled LCD interventions in the management of people with prediabetes or T2DM. Data sources included Medline, Embase, CINAHL, and Web of Science. Randomised (RCTs) or non-randomised (non-RCTs) controlled trials investigating the effect of technology-enabled LCDs (<130 g/day) or very low carbohydrate diets (VLCDs < 50 g/day) on glycosylated haemoglobin A1c (HbA1c) for at least three months and published in English between 2009 and 2023 were included. Risk of bias assessment, data extraction, and synthesis were conducted using standard tools and procedures. Six studies (two RCTs and four non-RCTs, total sample, *n* = 1519) were identified and included in the SLR. Two studies examining LCDs reported significant reductions in mean HbA1c (0.4% and −1.2%) and weight loss (−3.8 kg and −7.5 kg) at one year. Three studies examining VLCDs reported significant reductions in mean HbA1c (−0.8% to −1.3%) and weight loss (−12 kg to −14 kg) up to two years. Technology-enabled LCD or VLCD interventions can be a novel approach in helping people with prediabetes or T2DM self-manage their condition and possibly achieve remission. However, further research is required to determine the sustainability, effectiveness, and safety of this approach.

## 1. Introduction

Type 2 diabetes mellitus (T2DM) is a progressive metabolic condition characterised by insulin resistance and pancreatic B-cell dysfunction, resulting in hyperglycaemia [1]. People normally progress from normal glucose levels to impaired glucose tolerance or impaired fasting glucose (known as prediabetes) and then to T2DM. T2DM is diagnosed when glycosylated haemoglobin (HbA1c) levels reach or exceed 6.5% [2]. People with prediabetes represent those at high risk for developing T2DM, and they are diagnosed with a HbA1c of 5.7−6.4% [3]. The diagnostic criteria for prediabetes [3] and T2DM [2] are presented in Table 1.

Type 2 diabetes mellitus (T2DM), which represents over 90% of all types of diabetes [4], affects approximately 537 million people globally, and is expected to increase to around 783.2 million people by 2045 [5], thus placing T2DM as a leading public health problem globally [6]. According to the International Diabetes Federation [7] in 2021, 541 million adults were reported to have prediabetes and were therefore at a high risk for developing T2DM.

Non-modifiable risk factors that can increase an individual’s susceptibility to T2DM include genetics [1], age [8], and gestational diabetes in women [9]. While some people may have a strong genetic predisposition for T2DM, the risk considerably increases under exposure to several modifiable lifestyle factors, including physical inactivity, poor diet quality, and smoking [8,10].

Obesity is an important clinical risk factor for T2DM, as these two conditions are strongly linked and referred to as diabesity [11]. Obesity is associated with chronic inflammation and insulin resistance [12] and has been considered to be the cause of up to 80% of T2DM cases [13].

Furthermore, central obesity is a main feature of the metabolic syndrome [14], which represents a cluster of metabolic abnormalities that include insulin resistance, hypertension, and dyslipidaemia [15] and is also associated with increased risk for developing T2DM [16].

T2DM can be prevented and managed through lifestyle changes, with the probability of prevention being greater when prediabetes is identified and managed in a timely manner [17]. Physical activity (PA) is considered a key component in the prevention and management of T2DM [18,19], since adequate PA levels have been associated with improved insulin sensitivity and a reduced risk of T2DM [20,21].

Dietary changes are also considered essential for glycaemic control in T2DM prevention and management [21]. Several dietary approaches have been tested, with calorie-restricted diets being among the most popular ones. The DiRECT trial assigned 157 participants to consume a calorie-restricted diet through diet replacement (825–853 kcal/day) for three to five months, followed by gradually reintroducing food (1–8 weeks) and providing ongoing support for long-term weight loss management. This study reported weight loss of 15 kg or more in 36 (24%) study participants, while T2DM remission (i.e., people with T2DM that reduced their HbA1c below 6.5% and sustained this reduced level for more than three months without glucose-lowering medications [21]), reached 46% at 12 months. However, the study also showed increased rates of withdrawals, with 25% of participants in the intervention group dropping out. Frequently reported adverse events that were listed as reasons for withdrawal included constipation, headaches, and dizziness [22]. These findings indicate that for some people a calorie-restricted diet may not be a feasible, sustainable, and practical long-term solution for T2DM management.

Diabetes Australia has acknowledged that LCDs can be an effective dietary intervention for T2DM management [21]. A LCD is defined as a diet providing less than 130 g of carbohydrates per day or <26% of total caloric intake, whereas a very low carbohydrate diet (VLCD) (also known as a ketogenic diet) provides 20 g to 50 g of dietary carbohydrates or <10% of total caloric intake [23]. Emerging evidence suggests that LCDs are an effective way to reduce body weight and HbA1c. In a prospective open-label randomised controlled trial (RCT), 66 participants with T2DM were assigned to a LCD or calorie-restricted diet and received five 30 min face-to-face nutrition education sessions over the trial period; at six months a 0.58 kg/m^2^ decrease in BMI and a 0.65% reduction in HbA1c were observed in the LCD group [24]. These findings are also supported by a six-month parallel-group RCT by Dorans et al. who randomly assigned 75 participants to a LCD. This study reported a 0.26% reduction in HbA1c as well as a significant six-month decrease in fasting blood glucose (−8.4 mg/dL) and body weight (−6.4 Kg) [25].

LCDs have also been shown to be effective in a primary care-based cohort study spanning eight years which included 186 people with T2DM. This study reported the results of a LCD as implemented within conventional one-on-one GP consultations and reported that those participants who reduced their intake of carbohydrates to less than 130 g/day achieved an average of 11 kg weight loss, 97% achieved better glycaemic control (average HbA1c decreased from 7.9% to 6.4%), and T2DM remission was observed in 51% of study participants within an average time period of 33 months [26].

In a recent systematic review and meta-analysis of 23 RCTs [27], which included 1357 participants and evaluated the effectiveness and safety of LCDs and VLCDs in T2DM, 18 studies showed that participants allocated to the LCD arm achieved greater weight loss (7.4 kg) and 17 studies reported a greater HbA1c reduction (0.47%) at six months. Furthermore, LCDs were more effective in reducing fasting blood glucose levels by 0.73 mmol/L compared to low-fat diets. Eight studies included in this systematic review and meta-analysis also reported results related to T2DM remission at six months and three at 12 months. Overall, based on moderate certainty evidence LCDs were associated with a 32% increase in T2DM remission at six months. The studies included in the systematic review mostly relied on standard nutrition care practices to deliver the intervention [27]. Despite the emerging evidence [28] indicating that the implementation of dietary interventions using digital tools can be quite effective in reducing HbA1c levels, there are very few studies examining the effectiveness of interventions combining LCDs with digital tools.

### Objective

Providing support to people with T2DM can be a challenge as the availability and accessibility of standard care (face-to-face) is not always guaranteed, especially for vulnerable population groups with disabilities and/or people living in remote areas. LCDs have been proven to be effective in the treatment of T2DM to reduce HbA1c, specifically over a six-month period. However, to our knowledge, there are no systematic reviews examining the combined effect of LCD interventions delivered through digital tools on the prevention or treatment of T2DM. It is also important to consider if LCD interventions utilising digital technologies can consistently reduce HbA1c and sustain this reduction in the long term.

The aim of this systematic literature review (SLR) was to identify and narratively synthesise the available evidence on the effectiveness of technology-enabled LCD interventions for the prevention or management of T2DM in adults.

## 2. Materials and Methods

The current SLR was conducted as per the criteria defined by the PRISMA 2020 statement [29]. The review protocol was registered with PROSPERO on 2 May 2023 (protocol registration ID CRD42023415626) and can be accessed via: https://www.crd.york.ac.uk/prospero/#myprospero.

### 2.1. Eligibility Criteria

The inclusion and exclusion criteria for this SLR were guided by PICO(S). The inclusion criteria were relevant to the populations (P), interventions (I), comparisons (C), outcomes (O), and study (S) designs included in this review [29]. Regarding the population, this review included clinical trials with adult (>18 years old) participants of any ethnicity diagnosed with prediabetes or T2DM. To meet the inclusion criteria for the SLR, included studies had to investigate technology-enabled LCDs (<26% calories from carbohydrates or <130 g/day) or VLCDs (<10% calories from carbohydrates or <50 g/day) interventions for a defined period of at least 12 weeks with glycosylated haemoglobin A1c (HbA1c) as the primary outcome. The technology-enabled interventions included in this review were described as any online digital health intervention (e.g., mobile phone and web-based) where participants received education and health information by direct interface with the internet. A minimum follow-up period of three months was specified as the minimum duration of the intervention, since this is the time duration for new red blood cells to be produced and HbA1c levels to change [30]. The SLR identified and synthesised evidence coming from randomised controlled and non-randomised trials written in English and published between 2009 and 2023. The year 2009 was selected because this was when digital health applications started becoming widely used [31]. Studies were excluded if they reported data on children and adolescents (<18 years). Inclusion and exclusion criteria are reported in Supplementary Table S1.

### 2.2. Search Strategy and Information Sources

A comprehensive search strategy was designed that accounted for differences in search terms within each database and was adapted to each individual database (the complete search strategy is available in the Supplementary Materials). MEDLINE/PubMed EMBASE/Ovid, CINAHL via EBSCO, and Web of Science databases were systematically searched between 7 April and 9 April 2023, to identify studies published in English. PICO (S) components were represented by subject headings and applicable key word search terms. Data syntax appropriate to each database was applied. Each concept heading and keyword term within the population, intervention, comparator, and outcome categories was combined using truncations and Boolean Operators (“OR” and “AND”). An example of the MeSH terms used for Medline/Ovid included “Diabetes Mellitus, type 2”, “Diet, Carbohydrate-Restricted” and “Glycosylated Haemoglobin”, key words included “carbohydrate-restricted diets”, “diabetes mellitus, type 2”, “pre-diabetes”, “mhealth” and “HbA1c”. Limits were applied across databases for language, age, and publication date. In addition to the database searches, forward citation tracking in Google Scholar, hand searching, and citation mining of reference lists (Web of Science) of included studies were undertaken. One author of an unpublished but completed RCT was also contacted for sharing data [32]. The current SLR analysed and presents primary and secondary outcome data identified from studies relevant to the review question. The primary outcome identified was HbA1c, while secondary outcomes included body mass index (BMI), body weight, and percentage of the participants achieving T2DM remission [21].

### 2.3. Study Selection 

**Figure 1:**
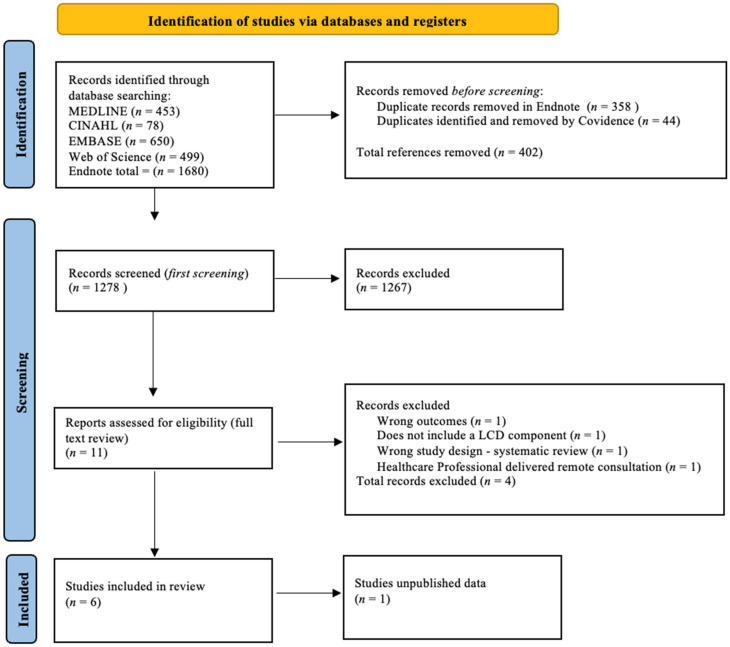
PRISMA flow diagram of included studies.

The systematic search of the above-mentioned databases was conducted by one author as per the SLR protocol registered with PROSPERO. Results were exported to Endnote version 20 [33], and 44 duplicates were removed based on the Bramer method of de-duplication [34]. All retrieved studies were imported to the Covidence systematic review software [35]. The titles and abstracts of the studies imported to Covidence were screened by one author, and unrelated studies were further removed. This first screening resulted in 11 studies for full text review. Two authors independently reviewed the full text based on the inclusion and exclusion criteria. No records with missing text were identified. The full-text review identified seven studies that were eligible for inclusion in the review. Where full-text versions of studies are unavailable, they were excluded. Disagreements were resolved through discussion between both authors, while a third author resolved inconsistent assessments by the first two authors. The final number of included records was decided by all reviewers. Reasons for exclusion were recorded and reported in Figure 1.

### 2.4. Data Collection Process and Data Items

A data extraction template was designed in Excel by one author based on the guidelines in the Cochrane Handbook for Systematic Reviews of Interventions [36]. The key categories to extract information from were relevant to the study design, participant characteristics, intervention, outcomes, and results. More specifically, the data extracted from the selected studies included: publication details (title, journal, publication year), author’s details (names, affiliations, conflict of interest and funding sources), study details (study design, start and end date, aim, country, setting, recruitment methods, blinding, randomisation, retention rate and statistical analysis), inclusion and exclusion criteria, study participants characteristics (sample size, age, gender, ethnicity), intervention (type of digital mode used, information on dietary intake, duration of the intervention), primary outcomes (HbA1c, mean (SD)), secondary outcomes (e.g., body weight (kg), mean difference (SD)), comparator details (characteristics of the control intervention where applicable), results, timepoint for follow-up and conclusion (key findings, strengths, and limitations). One author piloted the extraction form on a subset of included studies, one author reviewed the pilot extraction, and the results of the initial pilot were also discussed to ensure consensus. Following consensus and the finalisation of the data extraction template, one author independently extracted all data from the selected studies.

### 2.5. Risk of Bias and Quality Assessment in Individual Studies 

The risk of bias in selected studies was assessed by two authors, using the Quality Criteria Checklist for Primary Research of the American Academy of Nutrition and Dietetics [37]. This checklist includes four “relevance” and 10 validity questions, while providing a stepwise process to assess the quality and rigour of primary research studies and evaluate the strengths and weaknesses of the studies selected for the SLR to determine their internal and external validity, reliability, and generalisability. An overall positive, neutral, or negative result was assigned to each study. Where studies received mostly “yes” to validity criteria questions (specifically questions 2, 3, 6, and 7), the overall study received a positive result (meaning the study addressed bias regarding inclusion/exclusion, generalisability, data collection, and analysis), which indicates a low risk of bias. Studies with six or more “no” responses to the checklist items received an overall negative result, indicating a high risk of bias. If responses to questions 2, 3, 6, and 7 were unclear or not met, this indicated that the study was not particularly strong, and the study was designated a neutral result. Unclear or missing data for conflict-of-interest statements, funding, and affiliations increased the risk of bias.

### 2.6. Summary Measures

To report on the effectiveness of digitally delivered LCD or VLCD interventions, the main outcome of interest in this SLR was the mean difference in HbA1c levels. More specifically, from the selected RCTs we extracted the mean difference and standard deviation (SD) of HbA1c between the intervention and control groups at baseline and at each follow-up time point (e.g., at 3, 6, and/or 12 months). From the selected non-RCTs (i.e., two before-and-after studies and two single-arm pre-post interventions), the mean changes in HbA1c levels from baseline to follow-up were extracted and incorporated in the relevant data extraction template. Regarding secondary outcomes, the mean difference and SD in body weight and BMI at different time points and/or the changes from baseline to follow-up were also extracted. Data on percentage retention and T2DM remission were also extracted and reported from those studies that provided this information. Where available, data on participant engagement and adherence to the intervention was also extracted from the selected studies. Further to the statistical significance (*p* < 0.05) of the mean differences between groups and/or the mean changes within groups, the interventions were considered effective also based on their clinical significance, which was defined as a reduction in HbA1c of ≥0.5% and/or weight loss of >5% from baseline.

### 2.7. Data Synthesis

Because of the heterogeneity of the selected study designs, the extracted data were narratively synthesised. However, to summarise the effectiveness of the interventions examined in the selected studies, we used the Synthesis without Meta-analysis (SWiM) in systematic reviews reporting guidelines [38], thus ensuring further standardisation in data synthesis and reporting. Key findings were synthesised in a structured manner based on subgroups (i.e., reported for RCTs and non-RCTs separately). Findings were interpreted and synthesised in relevance to the SLR question.

### 2.8. Meta-Bias/Risk of Bias across Studies 

To control for meta-bias, a well-designed comprehensive search strategy was developed and tested by a senior librarian at La Trobe University. This process facilitated a more targeted identification of relevant studies (published and unpublished) and also served as a measure to control for publication bias [39]. The inclusion and exclusion criteria were pre-defined, which ensured that the studies were chosen based on precise eligibility requirements controlling for selection bias [39]. Two authors independently screened the full text of the selected studies. The use of the data extraction form also ensured standardised data extraction across studies. Regarding other measures that were taken to reduce the risk of bias, each study was individually assessed by two authors. The author of a recently completed study was contacted to explore the possibility of sharing preliminary results, and other publications from the same study and study protocols (where available) were reviewed to retrieve missing data (where applicable) or confirm unclear methodologies.

## 3. Results

### 3.1. Study Selection

The search identified an initial number of 1680 studies, from which 358 duplicates were removed in Endnote using a de-duplication removal process, thus leaving 1322 studies that were then imported to Covidence online software [35]. Covidence removed a further 44 studies as duplicates, leaving a total of 1278 studies for title and abstract screening. One author applied the inclusion and exclusion criteria, completed the first screening of titles and abstracts, and excluded 1267 studies. Eleven articles were identified for full-text review, which was conducted independently by two authors. Following this second, more thorough screening, four additional studies were further excluded. The main reasons for exclusion were the wrong outcome or study design, not including LCD as an intervention component, and being delivered by health care professionals instead of digital tools. This information is summarised in the PRISMA flow diagram in Figure 1.

There was no disagreement over the exclusion of these studies between the two independent reviewers. Seven studies met the inclusion criteria and were further processed for data extraction. However, six studies were included in this SLR [13,17,40,41,42,43], as one record was a study protocol, and although the corresponding author was contacted to provide us with unpublished data on the intervention’s effectiveness, unfortunately the requested data could not be made available [32]. Complementary data for each of the included studies was obtained from relevant online sources, where available. Of the selected studies, two were RCTs (a parallel-group pilot RCT [42] and a single-blind pilot RCT [17]), and four non-RCT studies (two before-and-after interventions [40,41] and two pre-post interventions [13,43]). All included studies implemented LCDs using a digital tool.

### 3.2. Study Characteristics

The six studies selected for the SLR provided results from a total of 1519 participants from three countries: the USA [40,41,42], the UK [13,43], and China [17]. Only adults with prediabetes and/or T2DM were represented in all studies, with an adult age range of >18 in one pre-post study [13] and one RCT [42], 18–99 years in one pre-post study [43], 21–65 in two before-and-after studies [40,41], and 30–80 years in one RCT [17]. The mean age of participants from all selected studies ranged from 53.0 years [42] to 56.1 years [43]. A summary of participants and study characteristics is available in Table 2 and Table 3.

#### 3.2.1. Recruitment, Sample Size and Follow-Up Period

Convenient sampling was used to recruit participants from only one general practice [13] or via referrals [40,41] and/or local advertisements [17,42,43]. The sample sizes also ranged from 25 participants [42] to 1000 participants [43] in two studies examining people with T2DM. Two other studies reported data from 349 participants with T2DM who were followed over an intervention period of one [41] and two years, respectively [40]. Chen et al. examined 100 participants with prediabetes [17], while Summers et al. examined 45 participants with prediabetes or T2DM [13]. One RCT had an even distribution of male and female participants [42], while another RCT included predominantly (i.e., 59.6%) female participants [17]. Similarly, more females were reported in three non-RCTs, i.e., 59% [43] and 68% [40,41], while only one non-RCT included more males (58%) than females [13]. Regarding ethnicity, in one RCT and two non-RCT studies most participants were white (58%, 87% and 90.4%, respectively) [13,42,43]. While participants in the other two non-RCT studies [40,41] were mostly white, these two studies only reported the percentage of African American participants. The duration of the intervention examined in the two RCTs was three [17] and eight months [42], respectively, while the non-RCTs had a total duration that ranged from 12 [13,41,43] to 24 months [40].

#### 3.2.2. Characteristics of Interventions

The RCT by Saslow et al. was digitally delivered over 16 weeks and included behavioural adherence strategies [42]. The lesson content was emailed to participants weekly for 16 weeks and then every two weeks for the last 16 weeks. Content included short video lessons, printable handouts, and links to online resources. The RCT by Chen et al. used the CAReNA mobile app to provide dietary recommendations twice over the three-month intervention period [17].

Two non-RCTs provided structured online education on LCD over 10 [43] and 12 weeks [13], respectively. These studies used a peer-supported and nutrition-focused app that focused on lifestyle changes and weight loss. Lesson content consisting of short video lessons, printable handouts, and links to online resources was emailed to study participants on a weekly basis, while study participants were also given access to online forums to self-select their goals.

Two non-RCTs used a digitally delivered intensive continuous care intervention (CCI) self-management program (Virta Health) [40,41]. Recorded education was provided weekly for 12 weeks, biweekly for 12 weeks, and monthly for six months. Additional supporting resources included guides, blog posts, and recipes, while the technology-enabled support provided to study participants also included telemedicine and health coaching.

#### 3.2.3. Carbohydrate Recommendations 

One RCT tested a VLCD (20 to 50g of carbohydrates) [42], while the second RCT [17] tested a LCD (70 g to 130 g of carbohydrates), aiming for a carbohydrate intake of 20–40 g per meal and 10 g per snack [17]. Two non-RCTs studies aimed for a VLCD (<30 g) as a means to induce nutritional ketosis (beta-hydroxybutyrate level of 0.5–3.0 mmol/L) [40,41], while the other two non-RCTs used a LCD (<130 g) focusing on portion control based on visual plate representations [13,43].

#### 3.2.4. Comparator Interventions

In both RCTs, the control groups received only standard diabetes diet management recommendations (i.e., based on the principles of a low-fat diet) [17,42]. The participants in the RCT by Saslow et al. also received online lessons around the American Diabetes Associations’ “Create Your Plate” diet for the first four weeks and then one lesson every four weeks for the remainder of the 32 weeks [42]. The control group in the two non-RCT studies [40,41] received usual care consisting of diabetes self-management, a low-fat diet and lifestyle recommendations.

### 3.3. Risk of Bias 

The risk of bias was assessed for each study individually using the Quality Criteria Checklist for Primary Research by the Academy of Nutrition and Dietetics [37]. Four studies received an overall positive score, which indicates a low risk of bias [17,40,41,42], while two studies received an overall neutral score [13,43], indicating a medium risk of bias. There were no incomplete data sets. Sample sizes were generally small and predominantly included female participants. The results from the assessment of the risk of bias for each one of the selected studies are summarised in Table 4. Regarding the risk of bias across studies, a potential source of this type of bias could be publication bias, considering that the preliminary unpublished results from a recently completed RCT could not be shared by the authors.

#### 3.3.1. Risk of Bias in Included RCTs

The RCT by Chen et al. only blinded outcome assessors [17], while in the RCT by Saslow et al. there was no blinding whatsoever [42]. This is something expected since blinding in dietary interventions is usually not feasible. In addition to bias stemming from non-blinding, the self-reported nature of health and dietary intake outcomes introduced recall bias in the two RCTs. Selection bias was also identified as sampling was convenient [17,42], thus resulting in non-representative samples, which negatively affects the generalisability of findings to the population [17,42].

#### 3.3.2. Risk of Bias in Included Non-RCTs

Two of the selected non-RCTs did not include control groups [13,43]. Selection bias was also identified, since convenience sampling was used in all selected non-RCTs, thus negatively affecting the representativeness and therefore the generalisability of the study findings [13,40,41,43]. Self-selection bias was also identified in two non-RCTs, since participants were not randomly allocated to treatment [40,41]. Self-reporting bias was identified in two of the selected non-RCTs [13,43] where participants self-reported their HbA1c test results and medication use. Recall bias was also identified in two non-RCTs [13,43], in which study participants relied on their memory to report their dietary intake. Selective reporting was identified in two non-RCTs where only baseline BMI data was reported [40,41], while bias related to conflict of interest due to sponsorship was identified in one non-RCT that did not report funding sources [13].

### 3.4. Results of Individual Studies 

#### 3.4.1. Summary Measures

The effectiveness of technology-enabled LCDs and VLCDs examined in the selected RCTs and non-RCTs was assessed via the mean between-group differences and/or within-group changes. All studies reported mean and SD values at baseline and follow-up in HbA1c (%) and body weight (kg) [13,17,40,41,42,43]. Four studies reported data on T2DM remission (%) [40,41,42,43], one study reported data on BMI (kg/m^2^) [17] and five studies reported data on retention [13,40,41,42,43]. Results for all primary and secondary outcomes are presented in Table 5 and Table 6.

#### 3.4.2. LCD and VLCD Effectiveness on HbA1c

Both RCTs showed a significant within-group reduction in HbA1c, with a significantly greater reduction (n = 12, *p* = 0.002) in HbA1c levels reported by Saslow et al. for the intervention group at 32 weeks that followed VLCD [42]. The same RCT also reported that more than half (55%) of study participants reduced their HbA1c to levels below 6.5%. The second RCT by Chen et al. reported a significant within-group decrease in HbA1c only in the intervention group, however, no significant difference was seen between groups, possibly due to the short (3-month) duration of the intervention [17].

Two non-RCTs reported statistically significant reductions in HbA1c, with participants in the CCI showing an average reduction in HbA1c levels of 1.3% at one year [41] and an overall reduction in HbA1c levels of −0.9% at two years [40] when following a VLCD.

One non-RCT that tested the LCD showed a significant reduction in HbA1c of 2.1% (n = 528, *p* < 0.001) in participants who engaged in all 10 modules of the intervention [43]. Similarly, a statistically significant mean reduction in HbA1c from 7.5% to 7.1% (*p* < 0.001) was observed in participants (29/45) who completed 75% of the lessons on a LCD [13], suggesting that better adherence to the intervention may result in a greater reduction in HbA1c.

#### 3.4.3. LCD and VLCD Effectiveness on Body Weight

In the two RCTs, those participants allocated to the intervention group had a significantly greater reduction in body weight of −12.7 Kg (n = 12, *p* < 0.001) over 32 weeks on a VLCD [42] and of −1.4 kg (*p* = 0.001) at 12 weeks on a LCD [17]. Two non-RCT studies (n = 262) achieved an average of −12% weight loss at one year [41], and a mean weight reduction of −10%, was reported at two years [40] on a VLCD. One non-RCT [43] reported a significant mean body weight change of −7.5 kg (*p* < 0.001) in participants (n = 528) who engaged in all 10 modules of the LCD intervention. Partial completers (n = 144) who engaged in four to nine modules showed a not statistically significant mean body weight change of −2.13 kg (*p* = 0.12) [43]. The non-RCT by Summers et al. reported a statistically significant mean body weight reduction of −3.8 kg (*p* < 0.001) in participants (29/45) who completed >75% of the lessons on a LCD [13]. Four studies showed various percentages of participants achieving weight loss of ≥5%; these were 90% at one year on a VLCD [42], 74% at two years on a VLCD [40], 46% at one year on an LCD [43], and 16% at one year on a LCD [13]. These results suggest that following a VLCD over a 12-month period may lead to greater weight loss when compared to LCDs.

#### 3.4.4. LCD and VLCD Effectiveness on Body Mass Index

Only one RCT reported results on BMI showing a significant between-group difference of 0.5 kg/m^2^ (*p* = 0.006) at three months [17]. Two non-RCTs reported only baseline data on BMI [40,41], while no BMI data was reported by three studies [13,42,43].

#### 3.4.5. LCD and VLCD Effectiveness on T2DM Remission

At 32 weeks the RCT by Saslow et al. reported that 55% of participants in the intervention group had reduced their HbA1c at levels below 6.5% [42]. In the non-RCT by Hallberg et al. 25% of participants reported diabetes remission at one year [41], while in the non-RCT by Athinarayanan et al. the percentage of remission was 17.6% at two years [40]. In the non-RCT by Saslow et al. 26% of participants (95/743) who had HbA1c levels above 6.5% at baseline reduced their HbA1c at levels below 6.5% without the use of glucose-lowering medication or while taking only metformin after 12 months, thus achieving T2DM remission [43].

#### 3.4.6. Retention and Engagement

The RCT by Saslow et al. reported a retention rate of 92% (11/12) in the intervention group compared to 54% (7/13) in the control group at 32 weeks [42]. The non-RCT by Hallberg et al. reported a retention rate of 83% (218/262) at one year [41], while at two years the retention rate dropped to 74% (194/262) [40]. In the non-RCT by Saslow et al. 71% (708/1000) of participants reported outcomes at 12 months, and of these, 51% (528/100) completed all modules [43]. In the non-RCT by Summers et al. 82% (37/45) of participants reported outcomes at 12 months, of which 64% (29/45) completed all 12 core modules [13].

#### 3.4.7. Outcome Reporting

In the RCT by Saslow et al. outcomes were measured using self-reported measures, 8% and 46% of participants in the intervention and control groups dropped out [42].

The RCT by Chen et al. used a validated questionnaire to collect self-reported dietary intake data from study participants [17]. In the non-RCT by Hallberg et al., participants self-reported the concentrations of their blood biomarkers using the relevant application [41]. In the non-RCT studies by Summers et al. and Saslow et al. study participants also self-reported their HbA1c levels [13,43]. Lastly, in the study by Saslow et al. 29% of study participants did not report outcomes at 12 months and their baseline data was used for the statistical analysis, assuming no change from baseline values [43]. Similarly, in the non-RCT study by Summers et al. the data from the last follow-up time point were used in the statistical analyses for the 18% of study participants that were lost to follow-up [13].

## 4. Discussion

To our knowledge, this is the first SLR of RCTs and non-RCTs to assess the effectiveness of technology-enabled LCDs or VLCDs in the prevention or treatment of T2DM. This systematic review highlights the limited body of evidence, specifically the available RCTs and non-RCTs examining the effect of digitally enabled LCD interventions in reducing HbA1c and body weight in people with prediabetes or T2DM.

### 4.1. Key Findings and Interpretation 

This SLR included and narratively synthesised evidence from six studies, including 1519 adult participants. Studies in this review compared LCDs or VLCDs using digital tools with standard care (using mainly low-fat diets). In a systematic review and meta-analysis including 56 trials (4937 participants) considering the effect of nine different dietary approaches (i.e., low fat, Mediterranean, high-protein, moderate carbohydrate, LCD, low glycaemic index or glycaemic load, vegetarian, Palaeolithic, and control diet) on glycaemic control; LCDs were reported as the most effective dietary approach for reducing HbA1c levels [44]. Three other systematic reviews further support this finding since they have also highlighted the effectiveness of LCDs in significantly reducing mean HbA1c levels in people with prediabetes or T2DM [45,46,47]. While various dietary approaches are used in glycaemic control and management of T2DM, our findings suggest that digitally delivered LCDs may be superior to standard care in terms of effectively lowering glucose levels.

The current SLR reported improved glycaemic control and significant weight loss in adults with prediabetes and T2DM following LCD or VLCD interventions delivered through digital tools. Subgroup evaluation revealed a greater reduction in mean HbA1c and body weight in studies examining VLCD compared to LCD. In this regard, three studies where participants were assigned to follow a VLCD for up to two years, reported reductions in HbA1c levels ranging from −0.8 to −1.3 and in body weight ranging from −12 kg to −14 kg [40,41,42]. However, two studies examining the effectiveness of LCD reported lower reductions (i.e., than the ones observed for VLCDs) in HbA1c levels that ranged from −0.4 to −1.2 and a weight reduction of −3.8 to −7.5 kg at one year [13,43].

The findings of the current SLR also indicate some clinically significant changes in both HbA1c levels and body weight, with most of the selected studies reporting reductions in HbA1c levels and weight losses of more than 0.5% and 5%, respectively [13,40,42,43]. This is expected since a modest weight loss of at least 5% has been reported to substantially improve glycaemic control [48]. The benefits of weight loss and the consequent glycaemic control can extend to several other health benefits. In this regard, a prospective study on people with T2DM showed a linear association between HbA1c levels and mortality, since for every 1% reduction in HbA1c level there was a parallel reduction in the risk of all-cause mortality by 21% [49].

Both RCTs included in this review showed superior results when LCDs [17] and VLCDs [42] delivered via digital tools were compared to standard dietary approaches (mainly low-fat diets) in terms of HbA1c reduction and weight loss. The VLCD used in the RCT by Saslow et al. produced the most significant decreases in HbA1c and body weight [42]. It is, however, important to note that the sample size included in this study was small, therefore the results should be interpreted with caution. Although findings such as those reported by Saslow et al. favour VLCD over LCD, a systematic review and meta-analysis [50] of RCTs reported that adherence to VLCDs appeared to be challenging, whereas adhering to LCDs seemed more feasible, with no apparent differences in clinical benefit observed between LCDs and VLCDs.

The second RCT by Chen et al. included in this SLR examined the effectiveness of LCD in adults with prediabetes and reported a significant between-group difference in postprandial glucose levels at the end of the intervention [17]. Even though the duration of the intervention in this RCT was only three months and did not include behavioural support [17], a significant within-group decrease in body weight, BMI, body fat mass, and all examined biomarkers from baseline to follow-up was reported. Compared to the control group that implemented a low-fat diet, the LCD group showed a favourable increase in questionnaire scores related to eating behaviour and exercise [17].

The current SLR suggests that higher intervention engagement is associated with a greater reduction in HbA1c, leading to a greater proportion of participants achieving T2DM remission. Most of the interventions included in this current SLR [13,40,41,42,43] reported retention rates ranging from 71% [43] to 92% [42]. As the design of most interventions was based on specific behavioural change theories and models, this possibly explains these high retention rates. This observation also suggests that by incorporating behaviour change techniques such as goal setting, peer support, and mindfulness training, adherence to interventions can be increased, thus resulting in a higher effectiveness of the intervention.

Five of the included studies ranging in intervention duration from eight months to two years included behavioural support, one RCT of three-month duration did not include behavioural support, nevertheless, all the included studies were effective in terms of having a positive impact on HbA1c levels and weight.

It is worth noting that the selected studies included participants who were primarily white, female, and older than 51. This probably indicates that populations of different ethnic, socio-economic, and age backgrounds have not been adequately represented in the available literature. A systematic review [51] suggests that various barriers exist that prevent adequate access to effective prevention or treatment initiatives for high-risk population groups, such as indigenous people in Australia [52]. Some barriers identified by Chopra et al. include the centre-based approach to delivery of care as opposed to home-based delivery, as well as transport and affordability of centre-based care [51]. The study also indicates that alternative approaches to primary care, such as the delivery of care in non-health centre settings or incorporating telecommunications strategies, could be used to reach vulnerable people affected by diabetes [51]. The use of digital tools to provide support and education on LCDs for T2DM management could achieve a wider reach and prove to be more effective than standard care in vulnerable populations with unmet needs.

### 4.2. Limitations and Strengths 

#### 4.2.1. Limitations

Limitations that were relevant to the selected studies include: non-randomised allocation of participants to treatment arms in the non-RCTs [13,40,41,43]; short intervention periods [17]; participants self-selecting their preferred treatment [17,40,41]; not measuring dietary intake data [42]; self-measured and self-reported data (i.e., weight, and medication changes) [42]; convenience sampling leading to selection bias and limiting the generalisability of the findings [13,40,41,42,43]; lack of ethnic diversity [40,41]; heterogeneity in study populations (prediabetes or T2DM or both) and diversity in studies regarding the inclusion of participants prescribed medication; medication use may impact on biochemical measures, and may confound the study findings [40,41,43]; digital literacy, which is an important factor that may affect engagement in a digitally delivered intervention was not assessed in any study.

Additional limitations that were relevant to the SLR include the narrative instead of statistical synthesis of results, it was therefore not feasible to pool data across the studies, and we were thus not able to apply the GRADE criteria tool or do a certainty assessment; we were unable to include data from an unpublished study as the corresponding author did not respond to our request; the inclusion of single group pre-post studies, which may have been compromised by selection bias, affecting the internal validity; although all included studies used digital tools as a delivery mode for their intervention, digital interventions was not consistent in all studies.

#### 4.2.2. Strengths

Strengths identified at the study level include the high reach of digital interventions and the large number of participants retained at eight months [42], 12 months [13,41,43] and two years [40]; data collected over various time points allowed for the assessment of how biomarkers change over time; findings showed that higher level of engagement leads to a greater reduction in HbA1c, body weight, and higher rates of T2DM remission.

Strengths identified at the review level include conducting this review according to the requirements of the PRISMA Statement [29]. A thorough search strategy was developed with the assistance of a senior librarian. Two reviewers conducted the full-text review, ensuring rigour and minimising bias; different levels of evidence were included, including RCTs that are considered the “gold standard” [53]; and the summary measures across the studies were similar which ensured effective comparison.

### 4.3. Implications and Recommendations 

The current SLR reported a positive link between the reduction in dietary carbohydrate intake and effective glycaemic control. In addition to the significant improvements in HbA1c and body weight, the current SLR showed that certain of the selected studies also resulted in T2DM remission, which is the goal of any intervention program to treat T2DM. In this regard, LCDs delivered through digital tools have the potential to become a cost-effective model of care in the hands of health professionals, thus providing a more solid basis for the prevention and treatment of T2DM. However, further research is required to provide more evidence on the long-term effectiveness and safety of digitally delivered LCDs, as well as their integration into primary health care.

## 5. Conclusions

Although T2DM continues to be a major public health concern, current mainstream recommendations for preventing and managing T2DM do not appear to be having a meaningful positive impact on the prevalence of this disease. The current SLR has shown that reliance on digital tools that educate and provide support to people with prediabetes or T2DM in self-managing their clinical condition based on the principles of LCD or VLCD can result in improved health, glycaemic control, and weight loss, which may result in T2DM remission. This novel approach has the potential to significantly improve health and quality of life in adults with prediabetes or T2DM, but further research is required to determine its long-term effectiveness, sustainability, and safety.

## Figures and Tables

**Table 1:** Diagnostic criteria for prediabetes and type 2 diabetes mellitus (T2DM).

	Prediabetes [3]	T2DM [2]
Fasting Blood Glucose (FBG)	6–6.9 mmol/L (100–125 mg/dL)	>7 mmol/L (126 mg/dL)
HbA1c	5.7−6.4% (39–46 mmol/mol)	>6.5% (48 mmol/mol)
Oral glucose tolerance test (2-h plasma glucose)	7.8–11.0 mmol/L (140–199 mg/dL)	>11.1 mmol/L (200 mg/dL)

**Table 2:** Characteristics of data synthesis of included randomised controlled trials (n = 2).

Author, Year, Study Design, Location	Sample Size N	Population Characteristics	Participants, N, Program Name Characteristics of Digital Intervention	Type of Diets Prescribed Intervention/Control	Primary Outcomes	Intervention/Follow-Up Period	Retention, Adherence	Risk of Bias
Saslow et al. (2017) Pilot RCT ^a^ Parallel-group, USA [42]	25	Overweight adults (>18) with T2DM ^b^, mean age 53.0 (IG ^c^), 58.2 (CG ^d^) years Patients receiving insulin (except for metformin) excluded	IG ^c^—N = 12, dietary instruction provided via weekly email lessons with lifestyle recommendations and mindfulness training CG ^d^—N = 13 dietary instructions provided via weekly emailed lessons. No lifestyle recommendations or mindfulness training included	IG ^c^: VLCD ^e^ ad libitum. (20–50 g/carbs per day), to induce nutritional ketosis CG ^d^: ADA ^f^ “create your plate” plan from the American Diabetic Association (low-fat diet)	HbA1c change	16-week intervention, 32-week follow-up	92% retention Drop out: IG ^c^: 8% (1/12) CG ^d^: 46% (6/13)	Positive No Blinding Self-report dietary intake
Chen et al. (2020) Pilot RCT ^a^ Single blinded China [17]	100	Adults (30–80 years) with prediabetes, mean age 54.9 (IG ^c^), 51.9 (CG ^d^) years. Patients taking medication excluded	IG ^c^ N = 57 Health management support service system. Mobile app (CAReNA). Receive LCD ^g^ dietary guidance CG ^d^—N = 43 Received early education in diabetes diet management	IG ^c^: LCD ^g^ (70–130 g/carbs per day). CG ^d^: Diabetes diet education. No LCD ^g^ intervention.	HbA1c change	3 months	-	Positive

^a^ RCT: randomized controlled trial, ^b^ T2DM: type 2 diabetes mellitus, ^c^ IG: intervention group, ^d^ CG: control group, ^e^ VLCD: very low carbohydrate diet, ^f^ ADA: American Diabetes Association, ^g^ LCD: low carbohydrate diet.

**Table 3:** Characteristics of data synthesis of included non-randomised controlled trials (n = 4).

Author, Year, Study Design, Location	Sample Size N	Population Characteristics	Participants, N, Program Name, Characteristics of Digital Intervention	Type of Diets Prescribed Intervention/Control	Primary Outcomes	Intervention/Follow-Up Period	Retention Adherence	Risk of Bias
Hallberg et al. (2018) Open-label, non-RCT ^a^ before-and-after study USA [41]	N = 349	Adults (21–65 years) with T2DM ^b^, mean age 53.7 years (CCI ^e^) Participants taking any diabetes medication included	N = 262 (CCI ^e^) IG ^c^: Virta Health, digitally delivered via the Virta app. CG ^d^: (N = 87) Usual diabetes care education by a dietitian	IG ^c^: VLCD ^f^ (<30 g/carbs per day) to induce nutritional ketosis CG ^d^: Usual diabetes diet (low fat)	HbA1c, body weight and medication use change	12 months	83% retention 25% remission	Positive selection bias; conflict of interest
Athinarayanan et al. (2019) Open-label non- RCT ^a^ before-and-after study USA [40]	N = 349	Adults (21–65 years) with T2DM ^b^, mean age 53.8 years (CCI ^e^) Participants taking any diabetes medication included	N = 262 (CCI ^e^) IG ^c^: Virta Health Digitally delivered via Virta app. CG ^d^: (N = 87) Usual diabetes care education by a dietitian	IG ^c^: VLCD ^f^ (<30 g/carbs per day) to induce nutritional ketosis CG ^d^: Usual diabetes diet (low fat)	HbA1c, body weight changes and retention	24 months	74% retention 17.6% remission	Positive selection bias; conflict of interest
Summers et al. (2021) Single arm pre-post prospective real-world study/intervention UK [13]	N = 45	Obese adults (>18) with T2DM ^b^ or prediabetes, mean age 54.8 years Participants taking any diabetes medication included	N = 45 The Low Carb Program—12 weeks digitally delivered. Included behavioural change techniques	LCD ^g^—(<130 g/carbs per day) based on visual plate representation	HbA1c and body weight change	12 months	82% retention	Neutral; no comparison; self-reporting outcome data; funding unclear
Saslow et al. (2018) Open-label single-arm, pre-post intervention USA [43]	N = 1000	Adults (>18) with T2DM ^b^, mean age 56.1 years. Participants taking any diabetes medication included	N = 1000 The Low Carb Program—10-week digitally delivered. Included behavioural change techniques	LCD ^g^—(<130 g/carbs per day) based on visual plate representation	HbA1c and body weight change	12 months	71% retention 26% remission	Neutral; no comparison; self-reporting outcome data

^a^ RCT: randomized controlled trial, ^b^ T2DM: type 2 diabetes mellitus, ^c^ IG: intervention group, ^d^ CG: control group, ^e^ CCI: continuous care intervention, ^f^ VLCD: very low carbohydrate diet, ^g^ LCD: low carbohydrate diet.

**Table 4:** Risk of bias assessment for included studies [38].

		Saslow et al. (2017) [42]	Chen et al. (2020) [17]	Hallberg et al. (2108) [41]	Athinarayanan et al. (2109) [40]	Saslow et al. (2108) [43]	Summers et al. (2021) [13]
	Overall rating	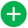	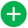	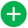	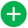	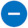	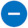
	Relevance Questions						
1	Would implementing the studied intervention or procedure (if found successful) result in improved outcomes for the patients/clients/population group? (NA for some Epi studies)	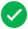	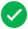	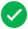	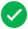	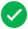	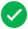
2	Did the authors study an outcome (dependent variable) or topic that the patients/clients/population group would care about?	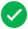	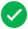	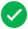	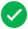	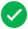	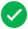
3	Is the focus of the intervention or procedure (independent variable) or topic of study a common issue of concern to dietetics practice?	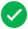	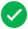	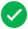	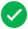	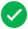	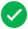
4	Is the intervention or procedure feasible? (NA for some epidemiological studies)	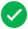	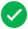	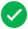	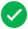	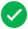	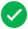
	Validity Questions						
1	Was the research question clearly stated?	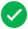	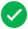	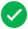	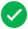	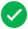	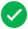
2	Was the selection of study subjects/patients free from bias?	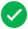	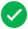	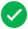	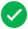	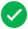	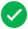
3	Were study groups comparable?	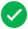	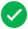	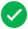	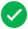	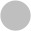	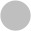
4	Was method of handling withdrawals described?	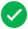	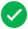	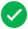	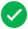	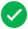	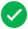
5	Was blinding used to prevent introduction of bias?	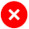	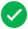	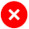	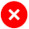	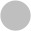	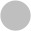
6	Were intervention/therapeutic regimens/exposure factor or procedure and any comparison(s) described in detail? Were intervening factors described?	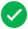	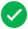	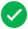	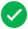	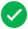	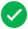
7	Were outcomes clearly defined and the measurements valid and reliable?	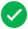	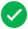	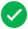	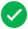	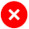	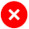
8	Was the statistical analysis appropriate for the study design and type of outcome indicators?	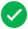	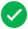	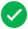	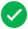	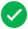	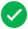
9	Are conclusions supported by results with biases and limitations taken into consideration?	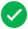	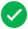	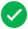	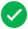	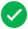	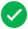
10	Is bias due to study’s funding or sponsorship unlikely?	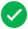	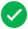	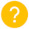	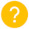	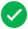	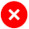

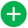
 Overall positive Risk of Bias Score; If most of the answers to the above validity question are “Yes” (including criteria 2, 3, 6, 7 and at least one additional “Yes”). 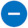 Neutral Risk of Bias Score; If the answers to the validity criteria question 2, 3, 6 and 7 do not indicate that the study is exceptionally strong. Negative Risk of Bias Score; If most (six or more) of the answers to the above validity question are “No”. 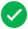 Yes, 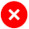 No, 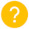 Unclear, 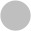 Not applicable.

**Table 5:** Significant clinical outcomes for the RCTs intervention group.

Author/Date/Reported Mean and Outcomes Measure	Baseline	Interim Follow-Up	Final Follow-Up	Within Group Change (*p* Value)	Between-Group Difference at Final Follow-Up (*p* Value)
Saslow et al. (2017) mean (SD ^b^) and mean EMM ^c^ (95% CI) [42]					
HbA1c ^a^ (%)	7.1 (0.4)	16 weeks = 6.2 (−1.1, −0.6)	32 weeks = 6.3 (−1.1, −0.6)	−0.8 (−1.1, −0.6)	−0.5 (−0.8, −0.2) 0.002
Weight (kg)	109.7 (24.9)	16 weeks = 101.4 (−11.9, −5.2)	32 weeks = 96.3 (−7.3, 1.3)	−12.7 (−16.1 to −9.2)	−9.6 (−14.0, −5.3) <0.001
Remission (%)	-	-	32 weeks = 55%	-	-
Chen et al. (2020) mean (SD), (95% CI ^e^) [17]					
HbA1c (%)	6.00 (0.39)	-	12 weeks = 5.77 (0.34)	<0.001	−0.10 (95% CI ^e^, 0.22, −0.03) 0.120
Weight (kg)	67.8 (11.7)	-	12 weeks = 65.6 (11.7)	<0.001	−1.4 (95% CI ^e^ 2.2, −0.6) <0.001
BMI ^d^ (kg/m^2^)	25.5 (2.9)	-	12 weeks = 24.8 (2.9)	<0.001	−0.5 (95% CI ^e^ 0.9, −0.2) 0.006

^a^ HbA1c: haemoglobin A1c (glycosylated haemoglobin), ^b^ Standard deviation, ^c^ EMM: estimated marginal means, ^d^ Body mass index, ^e^ Confidence intervals. Data were considered statistically significant if *p* < 0.05.

**Table 6:** Significant clinical outcomes for non-RCTs.

Author/Date/Reported Mean and Outcomes Measure	Baseline	Interim Follow-Up	Final Follow-Up	Within Group Change Mean Change (SD) (*p* Value)
Hallberg et al. (2018) mean (SD) [41]				
HbA1c ^a^ (%) Completers (n = 204)	7.5 (1.4)	-	1-year = 6.20 (0.9)	−1.3 (1.3) <10^−16^
Weight (kg), mean (SD) Completers (n = 184)	115.4 (24.6)	-	1-year = 101.2 (22.06)	−14.2 (10.3) <10^−16^
Remission			1-year = 25%	
Athinarayanan et al. (2019) mean (SD) [40]				
HbA1c ^a^ (%)	7.6 (1.5)	1-year = 6.2 (0.9)	2-years = 6.6 (1.3)	−0.9 (±0.1) 0.01
Weight (kg)	116.5 (25.9)	1-year = 101.1 (22.2)	2-years = 102.5 (25.2)	−11.9 ± 0.9 4.6 × 10^−26^
Remission (%)	-	-	2-years = 17.6%	-
Summers et al. (2021) mean (SD) [13]				
HbA1c ^a^	7.5 (1.65)	-	1-year = 7.1 (1.44)	−0.4% <0.001
Weight (kg) Completers (>9 modules)	91.5 (15.1)	-	1-year = 87.7 (14.5)	−3.8 (2.4) <0.001
Saslow et al. (2018) mean (SD) [43]				
HbA1c ^a^ (%) Completers (n = 528)	7.4 (1.8)	-	1-year = 6.2 (1.2)	−1.2 (1.4) <0.001
Weight (kg), Completers (n = 528)	88.8 (22.2)	-	1-year = 81.4 (17.9)	−7.5 (12.6) <0.001
Remission	-	-	1-year = 26.2%	-

Standard deviation (SD), ^a^ HbA1c: glycated haemoglobin, *p* value within the group (intervention) change and between groups (intervention and control). Data were considered statistically significant if *p* < 0.05.

## Data Availability

Not applicable.

## Supplementary Materials

The following supporting information can be downloaded at: https://www.mdpi.com/article/10.3390/nu15204362/s1, Table S1: PICO(S) Summary of Eligibility Criteria; Table S2: Medline/Ovid search strategy; Table S3. CINAHL/EBSCO search strategy; Table S4: EMBASE/Ovid search strategy; Table S5: Web of Science search strategy.
