# Supplementary material for: Effectiveness of Technology-Enabled, Low Carbohydrate Dietary Interventions, in the Prevention or Treatment of Type 2 Diabetes Mellitus in Adults: A Systematic Literature Review of Randomised Controlled and Non-Randomised Trials
Source: Nutrients. 2023 Oct 13;15(20):4362. doi: 10.3390/nu15204362 (PMC10609579; doi:10.3390/nu15204362)
Supplement: Supplementary file 1 [file nutrients-15-04362-s001.zip › nutrients-2598793-supplementary.pdf]

Effectiveness of Technology-Enabled, Low Carbohydrate Dietary Interventions, in the Prevention or Treatment of Type 2 Diabetes Mellitus in Adults: A Systematic Literature Review of Randomised Controlled and Non-Randomised Trials

Author Information:

Bernice Rozemai Jooste, Despina Kolivas, Peter Brukner and George Moschonis *

School of Allied Health, Human Services & Sport, La Trobe University,
Bundoora 3086, Australia; 18790557@students.latrobe.edu.au (B.R.J.); d.kolivas@latrobe.edu.au (D.K.); p.brukner@latrobe.edu.au (P.B.)
* Correspondence: g.moschonis@latrobe.edu.au

Supplementary Material

Supplementary Table S1: PICO(S) Summary of Eligibility Criteria ……………………….. 1
Supplementary Table S2: Medline/Ovid search strategy …………………………………....3
Supplementary Table S3: CINAHL/EBSCO search strategy ………………………………..5
Supplementary Table S4: EMBASE/Ovid search strategy…………………………………..7
Supplementary Table S5: Web of Science search strategy…………………………………...9

Supplementary Table S1: PICO(S) Summary of Eligibility Criteria

	Eligibility Criteria	
**Criteria**	**Inclusion Criteria**	**Exclusion Criteria**
**Population**	Adults with T2D (>18 years)<br>Adults with prediabetes (>18 years)	Children and adolescents (<18 years)<br>Animal studies
**Intervention**	Any digital health intervention (e.g., mobile phone and tablet apps, websites)<br>Studies investigating LCDs (<26% calories from carbohydrates or <130 g/day) or VLCDs (10% calories from carbohydrates or <50 g/day)<br>Technology enabled Low Carbohydrate Dietary (LCD) Intervention	Digital health interventions consisting of only tele-counselling or tele-monitoring.<br>Any dietary approach that does not fit the definition of LCD
**Comparison**	Non-technology enabled interventions, standard or usual care e.g., face- to-face interventions, diabetic educators, health education or promotion. Different dietary approaches (e.g., Low-fat diet)	Comparators that include digital health interventions
**Outcome**	Primary Outcome<br>Glycosylated haemoglobin (HbA_{1c})<br>Secondary Outcome<br>BMI<br>Body weight (BW)<br>% of participants achieving remission of T2D (HbA_{1c} <6.5 for at least 3 months and without any use of medication	Not reporting HbA_{1c}
**Study Design**	Randomised controlled trials and non-randomised trials	Observational, cross-sectional, case studies, case series, case-control, systematic reviews, meta-analyses.

Note. HbA_{1c} = Haemoglobin A1c, BMI = Body Mass Index, LCDs = low carbohydrate diets, VLCD = very low carbohydrate diets

Supplementary Table S2: Medline/Ovid search strategy

Date of search: 9/04/2023

	#	Search String
Population	1	**MeSH**:<br>exp Diabetes Mellitus, Type 2 +
	2	**Keywords:**<br>((diabet* adj2 "type 2") or "type ii")
	3	(T2D OR T2DM) OR ("Type 2" or "Type ii") OR (TIID OR TIIDM)
	4	(Pre-diabet* OR prediabet*)
	5	1 OR 2 OR 3 OR 4
Intervention	6	**MeSH:**<br>exp Diet, Carbohydrate-Restricted +
	7	**Keywords:**<br>(diet* adj2 carb*) OR (carb* adj2 restrict*) OR (carb* adj2 low) OR "very low carb*"
	8	(LCD OR VLCD OR VLCKD)
	9	(keto adj2 diet*) OR (Keto* OR KD)
	10	"Nutritional ketosis"
	11	("low* carb*" adj2 "health* fat*") OR LCHF
	12	("high protein" adj2 low* carb*) OR "high* protein*" OR "Atkins diet*"
	13	6 OR 7 OR 8 OR 9 OR 10 OR 11 OR 12
Intervention	14	**MeSH:**<br>exp Telemedicine +
	15	**Keywords:**<br>(tele* adj2 health*) OR (*Telecommunications OR Computers OR handheld OR microcomputers OR "User-computer interface" OR Internet)
	16	"Health education" OR "virtual health" OR "virtual medicine" OR "virtual care" OR "virtual intervention" OR "remot* health" OR "distal technology*" OR "remote monitor*" OR "health services accessibility" OR (therapy adj2 "computer assisted") OR (delivery adj2 "health care")
	17	("Smart-phone*" OR smartphone* OR iphone OR android) OR "*social networking" OR podcast* OR "text messag*" OR app OR apps OR "virtual reality" OR ipad* OR "tablet comput*" OR "tablet device*" OR "instant messag*" OR SMS* OR whatsapp OR Youtube* OR "video gam*" OR "interactive computer model"
	18	(m-health* OR mhealth* OR "Mobile health" OR "Mobile app*" OR "Mobile application*") OR (e-health* OR ehealth* OR "electronic health*" OR e-technolog* OR etechnolog* OR "electronic system*") OR ("digital health*" OR "digital technolog*") OR (e-learning* OR elearning* OR e-medicine* or emedicine* or e-therap* or etherap*) OR ("health information technolog*" OR "info* tech*" OR e-support*)
	19	((online OR "online intervention" OR on-line OR digital* OR electronic OR computer* OR software or internet* OR web or website* OR technology-based OR interactiv* OR telecommunicat* OR "information and communication technolog*" OR ICT) OR (support OR "self-help" OR chat OR communicat* OR "self care" OR "self manag*" OR "self-manag*" OR "self efficac*" OR selfefficac* OR "self-monitor*" OR intervention* OR education* OR training OR learning OR teaching OR "health information*" OR "information service*" OR lifestyle* OR "life style*" OR motivat* OR healthcare OR "health care" OR "health promotion*" OR "home health*" OR "home care"))

(Continues)

Table S2 (Continued)

	#	**Search String**
**Intervention**	20	**Keywords:**<br>"Internet based" OR "app* based" OR Web-based OR "technology-assisted" OR webcast* OR "lifestyle app*" OR "Computer-assisted" OR "Computer-based " OR "Computer interface"
	21	(glucose adj2 app*) OR "continuous glucose monitoring" OR CGM OR"health monitor* app*"
	22	14 OR 15 OR 16 OR 17 OR 18 OR 19 OR 20 OR 21
**Outcome**	23	**MeSH:**<br>exp Glycated hemoglobin +
	24	**Keywords:**<br>"Glycosylated h?emoglobin" OR ("H?emoglobin A" OR "H?emoglobin A1c") OR (HbA1c OR A1c) OR "Glyc* control" OR Hyperglyc* OR "Blood glucose level" OR BGL OR "blood sugar level" OR BSL OR "Fasting blood glucose" OR FBG
	25	**MeSH:**<br>exp Weight Loss +
	26	**Keywords:**<br>Weight-loss* OR (reduc* adj2 weight) OR (weight adj2 loss*) OR "Body mass index" OR BMI OR (waist adj2 circumference)
	27	23 OR 24 OR 25 OR 26
**Study Design**	28	**RCT Filter:**<br>Randomized controlled trial.pt.
	29	Controlled clinical trial.pt.
	30	Randomized.ab.
	31	Placebo.ab.
	32	Drug therapy.fs.
	33	Randomly.ab.
	34	Trial.ab.
	35	Groups.ab.
	36	28 OR 29 OR 30 OR 31 OR 32 OR 33 OR 34 OR 35
	37	exp animals/ not humans.sh.
	38	36 NOT 37
	38	5 AND 13 AND 22 AND 27 AND 36
	39	Limit 91 to English language and yr="2009 – 2023"
	40	Limit 92 to ("all adult (19 plus years)" or "(young adult (19 to 24 years)" or "Adult (19-44 years)" OR "young adult and adult (19-24 and 19-44)" or "middle age (45 to 64 years)" or "(middle aged (45 plus years)" or "all aged (65 and over)" or "aged (80 and over)"

NOTE. exp = explode MeSH, + is explode results (include all subheadings); adj2 (words adjacent to each other in any order within 2 words), "… " (exact phrase searching), * Truncation (to expand spelling and endings of words)? wildcards (adds one character),. ab (abstract word), .pt (publication type), fs (floating subheading), "OR" – includes either both, the one or the other term), "AND" – includes both terms.

Supplementary Table S3: CINAHL/EBSCO search strategy

Date of search: 9/04/2023

	#	Search String
**Population**	1	**MeSH**:<br>MH Diabetes Mellitus, Type 2 +
	2	**Keywords:**<br>((diabet* N2 "type 2") or "type ii")
	3	(T2D OR T2DM) OR ("Type 2" or "Type ii") OR (TIID OR TIIDM)
	4	(Pre-diabet* OR prediabet*)
	5	1 OR 2 OR 3 OR 4
**Intervention**	6	**MeSH:**<br>MH Diet, Carbohydrate-Restricted +
	7	**Keywords:**<br>(diet* N2 carb*) OR (carb* N2 restrict*) OR (carb* N2 low) OR "very low carb*"
	8	(LCD OR VLCD OR VLCKD)
	9	(keto N2 diet*) OR (Keto* OR KD)
	10	"Nutritional ketosis"
	11	("low* carb*" N2 "health* fat*") OR LCHF
	12	("high protein" N2 low* carb*) OR "high* protein*" OR "Atkins diet*"
	13	6 OR 7 OR 8 OR 9 OR 10 OR 11 OR 12
**Intervention**	14	**MeSH:**<br>MH Telemedicine +
	15	**Keywords:**<br>(tele* N2 health*) OR (*Telecommunications OR Computers OR handheld OR microcomputers OR "User-computer interface" OR Internet)
	16	"Health education" OR "virtual health" OR "virtual medicine" OR "virtual care" OR "virtual intervention" OR "remot* health" OR "distal technology*" OR "remote monitor*" OR "health services accessibility" OR (therapy N2 "computer assisted") OR (delivery N2 "health care")
	17	("Smart-phone*" OR smartphone* OR iphone OR android) OR "*social networking" OR podcast* OR "text messag*" OR app OR apps OR "virtual reality" OR ipad* OR "tablet comput*" OR "tablet device*" OR "instant messag*" OR SMS* OR whatsapp OR Youtube* OR "video gam*" OR "interactive computer model"
	18	(m-health* OR mhealth* OR "Mobile health" OR "Mobile app*" OR "Mobile application*") OR (e-health* OR ehealth* OR "electronic health*" OR e-technolog* OR etechnolog* OR "electronic system*") OR ("digital health*" OR "digital technolog*") OR (e-learning* OR elearning* OR e-medicine* or emedicine* or e-therap* or etherap*) OR ("health information technolog*" OR "info* tech*" OR e-support*)
	19	((online OR "online intervention" OR on-line OR digital* OR electronic OR computer* OR software or internet* OR web or website* OR technology-based OR interactiv* OR telecommunicat* OR "information and communication technolog*" OR ICT) OR (support OR "self-help" OR chat OR communicat* OR "self care" OR "self manag*" OR "self-manag*" OR "self efficac*" OR selfefficac* OR "self-monitor*" OR intervention* OR education* OR training OR learning OR teaching OR "health information*" OR "information service*" OR lifestyle* OR "life style*" OR motivat* OR healthcare OR "health care" OR "health promotion*" OR "home health*" OR "home care"))

(Continues)

Table S3 (Continued)

	#	**Search String**
**Intervention**	20	**Keywords:**<br>"Internet based" OR "app* based" OR Web-based OR "technology-assisted" OR webcast* OR "lifestyle app*" OR "Computer-assisted" OR "Computer-based " OR "Computer interface"
	21	(glucose N2 app*) OR "continuous glucose monitoring" OR CGM OR"health monitor* app*"
	22	14 OR 15 OR 16 OR 17 OR 18 OR 19 OR 20 OR 21
**Outcome**	23	**MeSH:**<br>MH Hemoglobin A +
	24	**Keywords:**<br>"Glycosylated h?emoglobin" OR ("H?emoglobin A" OR "H?emoglobin A1c") OR (HbA1c OR A1c) OR "Glyc* control" OR Hyperglyc* OR "Blood glucose level" OR BGL OR "blood sugar level" OR BSL OR "Fasting blood glucose" OR FBG
	25	**MeSH:**<br>MH Weight Loss +
	26	**Keywords:**<br>Weight-loss* OR (reduc* N2 weight) OR (weight N2 loss*) OR "Body mass index" OR BMI OR (waist N2 circumference)
	27	23 OR 24 OR 25 OR 26
**Study Design**	28	**RCT Filter:**<br>TX random* OR factorial* OR placebo* OR assign* OR allocat* OR crossover*
	29	TX "cross over"
	30	TX trial and TX (control* OR comparative)
	31	TX (blind* OR mask*) and TX (single OR double OR triple OR treble)
	32	TX "treatment arm"
	33	TX "control group*"
	34	TX phase and TX (three OR III)
	35	TX versus OR vs
	36	(MH "Clinical Trials+")
	37	(MH "Random Assignment")
	38	(MH "Quantitative Studies")
	39	(MH "Placebos")
	40	TX rct
	41	S28 OR S29 OR S30 OR S31 OR S32 OR S33 OR S34 OR S35 OR S36 OR S37 OR S38 OR S39 OR S40
	42	5 AND 13 AND 22 AND 27 AND 41
	43	Limit to English language
	44	Limit Published Date: 2009/0/101-2023
	45	Limit to Adult (19-44 years)" OR (middle aged 45-64) or aged 65+ years OR "aged (80 and over)"

Note. Cinahl / database coverage: 2009 to present, MH = MeSH, + is explode results (include all subheadings); N2 (finds the words if they are within two words of one another regardless of order), "… " (exact phrase searching), * Truncation (to expand spelling and endings of words),? wildcards (adds one character), .ab (abstract word), .pt (publication type), fs (floating subheading), "OR" – includes either both, the one or the other term), "AND" – includes both terms.

Supplementary Table S4: EMBASE/Ovid search strategy

EMBASE/Ovid (database coverage 2009-2023)

	#	**Search String**
**Population**	1	**MeSH**:<br>Exp non-insulin dependent diabetes mellitus +
	2	**Keywords:**<br>((diabet* adj2 "type 2") or "type ii")
	3	(T2D OR T2DM) OR ("Type 2" or "Type ii") OR (TIID OR TIIDM)
	4	(Pre-diabet* OR prediabet*)
	5	1 OR 2 OR 3 OR 4
**Intervention**	6	**MeSH:**<br>exp low Carbohydrate diet +
	7	**Keywords:**<br>(diet* adj2 carb*) OR (carb* adj2 restrict*) OR (carb* adj2 low) OR "very low carb*"
	8	(LCD OR VLCD OR VLCKD)
	9	(keto adj2 diet*) OR (Keto* OR KD)
	10	"Nutritional ketosis"
	11	("low* carb*" adj2 "health* fat*") OR LCHF
	12	("high protein" adj2 low* carb*) OR "high* protein*" OR "Atkins diet*"
	13	6 OR 7 OR 8 OR 9 OR 10 OR 11 OR 12
**Intervention**	14	**MeSH:**<br>exp Telemedicine +
	15	**Keywords:**<br>(tele* adj2 health*) OR (*Telecommunications OR Computers OR handheld OR microcomputers OR "User-computer interface" OR Internet)
	16	"Health education" OR "virtual health" OR "virtual medicine" OR "virtual care" OR "virtual intervention" OR "remot* health" OR "distal technology*" OR "remote monitor*" OR "health services accessibility" OR (therapy adj2 "computer assisted") OR (delivery adj2 "health care")
	17	("Smart-phone*" OR smartphone* OR iphone OR android) OR "*social networking" OR podcast* OR "text messag*" OR app OR apps OR "virtual reality" OR ipad* OR "tablet comput*" OR "tablet device*" OR "instant messag*" OR SMS* OR whatsapp OR Youtube* OR "video gam*" OR "interactive computer model"
	18	(m-health* OR mhealth* OR "Mobile health" OR "Mobile app*" OR "Mobile application*") OR (e-health* OR ehealth* OR "electronic health*" OR e-technolog* OR etechnolog* OR "electronic system*") OR ("digital health*" OR "digital technolog*") OR (e-learning* OR elearning* OR e-medicine* or emedicine* or e-therap* or etherap*) OR ("health information technolog*" OR "info* tech*" OR e-support*)
	19	((online OR "online intervention" OR on-line OR digital* OR electronic OR computer* OR software or internet* OR web or website* OR technology-based OR interactiv* OR telecommunicat* OR "information and communication technolog*" OR ICT) OR (support OR "self-help" OR chat OR communicat* OR "self-care" OR "self manag*" OR "self-manag*" OR "self efficac*" OR selfefficac* OR "self-monitor*" OR intervention* OR education* OR training OR learning OR teaching OR "health information*" OR "information service*" OR lifestyle* OR "life style*" OR motivat* OR healthcare OR "health care" OR "health promotion*" OR "home health*" OR "home care"))

(Continues)

Table S4 (Continued)

	#	**Search String**
**Intervention**	20	**Keywords:**<br>"Internet based" OR "app* based" OR Web-based OR "technology-assisted" OR webcast* OR "lifestyle app*" OR "Computer-assisted" OR "Computer-based " OR "Computer interface"
	21	(glucose adj2 app*) OR "continuous glucose monitoring" OR CGM OR"health monitor* app*"
	22	14 OR 15 OR 16 OR 17 OR 18 OR 19 OR 20 OR 21
**Outcome**	23	**MeSH:**<br>exp Glycosylated hemoglobin +
	24	**Keywords:**<br>"Glycosylated h?emoglobin" OR ("H?emoglobin A" OR "H?emoglobin A1c") OR (HbA1c OR A1c) OR "Glyc* control" OR Hyperglyc* OR "Blood glucose level" OR BGL OR "blood sugar level" OR BSL OR "Fasting blood glucose" OR FBG
	25	**MeSH:**<br>Exp body weight Loss +
	26	**Keywords:**<br>Weight-loss* OR (reduc* adj2 weight) OR (weight adj2 loss*) OR "Body mass index" OR BMI OR (waist adj2 circumference)
	27	23 OR 24 OR 25 OR 26
**Study Design**	28	**RCT Filter:**<br>(random* or factorial* or placebo* or assign* or allocat* or crossover*).tw.
	29	(cross adj over*).tw.
	30	(trial* and (control* or comparative)).tw.
	31	((blind* or mask*) and (single or double or triple or treble)).tw.
	32	(treatment adj arm*).tw.
	33	(control* adj group*).tw.
	34	(phase adj (III or three)).tw.
	35	(versus or vs).tw.
	36	rct.tw.
	37	Crossover Procedure/
	38	DOUBLE BLIND PROCEDURE/
	39	SINGLE BLIND PROCEDURE/
	40	RANDOMIZATION/
	41	PLACEBO/
	42	exp Clinical Trial/
	43	PARALLEL DESIGN/
	44	LATIN SQUARE DESIGN/
	45	28 OR 29 OR 30 OR 31 OR 32 OR 33 OR 34 OR 35 OR 36 OR 37 OR 38 OR 39 OR 40 OR 41 OR 42 OR 43 OR 44
	46	exp ANIMAL/ or exp NONHUMAN/ or exp ANIMAL EXPERIMENT/ or exp ANIMAL MODEL/
	47	exp HUMAN/
	48	46 NOT 47
	49	45 NOT 48
	50	5 AND 13 AND 22 AND 27 AND 45
	51	Limit to English language
	52	Limit Published Date: 2009/0/101-2023
	53	Limit to (Adult < 18 TO 64 years > or aged <65+ years>)

NOTE. exp = explode MeSH, + is explode results (include all subheadings); adj2 (words adjacent to each other in any order within 2 words), "… " (exact phrase searching), * Truncation (to expand spelling and endings of words)? wildcards (adds one character), . ab (abstract word), .pt (publication type),,fs (floating subheading), “OR” – includes either both, the one or the other term), “AND” – includes both terms.

Supplementary Table S5: Web of Science search strategy

Date of search: 9/04/2023

	#	**Search**
**Population**	1	Topic (TS):<br>TS=("Diabetes mellitus, type 2" OR "Type 2 diabet*" OR "Type 2 diabetes mellitus" OR "Diabetes mellitus type ii" OR T2D OR T2DM OR "type II" OR **"Type ii"** ORTIID OR TIIDM OR **"non-insulin dependent diabetes mellitus" OR** "Diabetes mellitus non-insulin dependent" OR "Diabetes mellitus adult onset" OR prediabetes OR diabetic* OR "pre-diabet*" OR "Pre-diabet*" OR prediabet*)
**Intervention**	2	Topic (TS):<br>TS=("Diet, Carbohydrate-Restricted" OR "Carbohydrate restricted" OR "Carbohydrate-restricted diets" OR "Dietary carbohydrat* restrict*" OR "Diets carbohydrate-restricted" OR "Diet low carbohydrate"<br>"Carbohydrate diet low" OR " low carb*" OR "diet* carb*" OR "carb* restrict*" OR "carb* low" OR "very low carb*" OR "LCD intervention*" OR LCD OR "carb* restrict* diet*" OR "Low carb* diet*" OR "Diets low carbohydrate" OR "Low carbohydrate diet*" OR "Very low carb*" OR "Very low carb* diet*" OR VLCD OR "Very low carb* keto* diet*" OR VLCKD OR LCD OR<br>"ketogenic" OR "keto* diet*" OR "keto*" OR KD OR "Nutritional ketosis" OR "Low carb* healthy fat" OR LCHF OR "High protein low* carb*" **OR** "high* protein*" OR "Atkins diet*")
	3	**#1 AND #2**
	4	**TS=**(tele* adj2 health*) OR (Telecommunications OR Computers OR handheld OR microcomputers OR "User-computer interface" OR Internet) OR "Health education" OR "virtual health" OR "virtual medicine" OR "virtual care" OR "virtual intervention" OR "remot* health" OR "distal technology*" OR "remote monitor*" OR "health services accessibility" OR (therapy "computer assisted") OR (delivery "health care") OR<br>("Smart-phone*" OR smartphone* OR iphone OR android) OR "social networking" OR podcast* OR "text messag*" OR<br>"app* based" OR app OR apps OR "virtual reality" OR ipad* OR "tablet comput*" OR "tablet device*" OR (m-health* OR mhealth* OR "Mobile health" OR "Mobile app*" OR "Mobile application*") OR (e-health* OR ehealth* OR "electronic health*") OR "instant messag*" OR SMS* OR whatsapp OR Youtube* OR "video gam*" OR "interactive computer model")

(Continues)

Table S5 (Continued)

	#	**Search**
**Intervention**	5	**TS=**(e-technolog* OR etechnolog* OR "electronic system*") OR ("digital health*" OR "digital technolog*") OR (e-learning* OR elearning*) OR (e-medicine* OR emedicine* OR e-therap* OR etherap*) OR ("health information technolog*" OR "info* tech*" OR e-support*) OR (online OR "online intervention" OR on-line OR digital* OR electronic OR computer* OR software OR internet* OR "Internet based" OR web OR website* OR technology-based OR interactiv* OR telecommunicat* OR "information and communication technolog*" OR ICT) OR (support OR "self-help") OR chat OR communicat* OR ("self care" OR "self manag*" OR "self-manag*" OR "self efficac*" OR selfefficac* OR "self-monitor*") OR intervention* OR education* OR training OR learning OR teaching OR "health information*" OR "information service*" OR lifestyle* OR "life style*" OR motivat* OR healthcare OR "health care" OR "health promotion*" OR "home health*" OR "home care" OR Web-based OR "technology-assisted" OR webcast* OR "lifestyle app*" OR "Computer-assisted" OR "Computer-based " OR "Computer interface" OR (glucose app*) OR "continuous glucose monitoring" OR CGM OR"health monitor* app*" OR "diabetes prevention")
	6	#4 OR #5
	7	#3 AND #6
**Outcome**	8	**TS=(**"Glycosylated h?emoglobin" OR ("H?emoglobin A" OR "H?emoglobin A1c") OR (HbA_{1c} OR A1c) OR "Glyc* control" OR Hyperglyc* OR "Blood glucose level" OR BGL OR "blood sugar level" OR BSL OR "Fasting blood glucose" OR FBG OR Body Weight-loss* OR "reduc* weight" OR "weight adj2 loss*" OR "Body mass index" OR BMI OR "waist adj2 circumference")
**Study Design**	9	**RCT Filter:** TS=(randomised OR randomized OR randomisation OR randomisation OR placebo* OR (random* AND (allocat* OR assign*)) OR (blind* AND (single OR double OR treble OR triple)))
	10	#7 AND #8 AND #9
	11	Limit publication years 2009-2023
	9	Limit o English language

NOTE. TS - MeSh and keywords were searched together, "OR" – includes either both, the one or the other term), "AND" – includes both terms. * Truncation (to expand spelling and endings of words).
